# Neuromuscular Properties of the Human Wrist Flexors as a Function of the Wrist Joint Angle

**DOI:** 10.3389/fbioe.2019.00181

**Published:** 2019-08-21

**Authors:** Martin Behrens, Florian Husmann, Anett Mau-Moeller, Jenny Schlegel, Eva-Maria Reuter, Volker R. Zschorlich

**Affiliations:** ^1^Institute of Sport Science, University of Rostock, Rostock, Germany; ^2^Centre for Sensorimotor Performance, School of Human Movement and Nutrition Sciences, The University of Queensland, Brisbane, QLD, Australia

**Keywords:** muscle length, voluntary activation, electrical stimulation, flexor carpi radialis, median nerve, twitch, post-activation potentiation, activity-dependent potentiation

## Abstract

The joint angle dependence of voluntary activation and twitch properties has been investigated for several human skeletal muscles. However, although they play a key role for hand function and possess a unique neural control compared to muscles surrounding other joint complexes, little is known about the wrist flexors innervated by the median nerve. Therefore, isometric voluntary and electrically evoked contractions of the wrist flexors were analyzed at three wrist joint angles (extension: −30°, neutral: 0°, flexion: 30°) to quantify the joint angle dependence of (i) voluntary activation (assessed via peripheral nerve stimulation and electromyography [EMG]), (ii) unpotentiated twitch torques, and (iii) potentiated twitch torques. Maximum voluntary torque was lower in extension compared to neutral and flexion. Although voluntary activation was generally high, data indicate that voluntary activation of the wrist flexors innervated by the median nerve was lower and the antagonist·agonist^−1^ EMG ratio was higher with the wrist joint in flexion compared to extension. Peak twitch torque, rate of twitch torque development, and twitch half-relaxation time increased, whereas electromechanical delay decreased from flexion to extension for the unpotentiated twitch torques. Activity-induced potentiation partly abolished these differences and was higher in short than long wrist flexors. Different angle-dependent excitatory and inhibitory inputs to spinal and supraspinal centers might be responsible for the altered activation of the investigated wrist muscles. Potential mechanisms were discussed and might have operated conjointly to increase stiffness of the flexed wrist joint. Differences in twitch torque properties were probably related to angle-dependent alterations in series elastic properties, actin-myosin interaction, Ca^2+^ sensitivity, and phosphorylation of myosin regulatory light chains. The results of the present study provide valuable information about the contribution of neural and muscular properties to changes in strength capabilities of the wrist flexors at different wrist joint angles. These data could help to understand normal wrist function, which is a first step in determining mechanisms underlying musculoskeletal disorders and in giving recommendations for the restoration of musculoskeletal function after traumatic or overuse injuries.

## Introduction

The isometric maximal voluntary torque (MVT) produced at a given joint angle depends, among other factors, on the voluntary activation of muscles by the central nervous system, the force-length properties of the muscle-tendon units (MTU) of interest as well as their variable moment arms and pennation angles. Several studies have shown that voluntary activation of muscles lowers with decreasing MTU length (Becker and Awiszus, 2001; Kubo et al., 2004; Bampouras et al., 2006; Prasartwuth et al., 2006; Doguet et al., 2017b; Lanza et al., 2017), while others have documented an increased or unchanged voluntary activation when MTUs get shorter (Suter and Herzog, 1997; Huber et al., 1998; Babault et al., 2003; Newman et al., 2003). Non-optimal stimulation techniques used for quantifying neural activation (e.g. insufficient number of stimuli as well as unfavorable type of stimulation and timing of the control twitch), the insensitivity of the measurement systems, the heterogeneity of the investigated participants, and the muscle group of interest might be responsible for the contradictory results. Two recently published studies compared voluntary activation of the knee extensors during isometric maximal voluntary contractions (MVC) at different joint angles and accounted for some methodological limitations mentioned above (Doguet et al., 2017b; Lanza et al., 2017). Their results support the previous observations of a decreased neural drive to muscles at short length (Becker and Awiszus, 2001; Kubo et al., 2004; Bampouras et al., 2006). It is thought that the physiological basis for this phenomenon is centered around the joint angle-dependent amount of afferent input from muscle spindles to the α-motoneuron pool and other mechanoreceptors such as ligament receptors, joint receptors, and Golgi tendon organs. The magnitude of this excitatory and inhibitory feedback might be altered due to changes in joint angle and thereby MTU length (Becker and Awiszus, 2001; Kubo et al., 2004). Studies investigating the joint angle dependence of voluntary activation have focused, e.g., on the knee extensors (Becker and Awiszus, 2001; Kubo et al., 2004; Doguet et al., 2017b; Lanza et al., 2017), plantar flexors (Kluka et al., 2016), dorsiflexors (Gandevia and McKenzie, 1988), elbow flexors (Gandevia and McKenzie, 1988; Prasartwuth et al., 2006), and finger abductors (Gandevia and McKenzie, 1988). To the best of our knowledge, this relationship has never been investigated for the wrist flexors innervated by the median nerve. This could be of particular interest, considering that the wrist flexors play a key-role for hand function, e.g., for carrying heavy objects and for tasks requiring forceful and/or rapid wrist flexions (Perry, 1978). Beyond that, the wrist flexors possess a unique neural control compared to muscles surrounding other joint complexes (Aymard et al., 1997; Wargon et al., 2006).

Although voluntary activation of muscles is considered as an important contributor to the torque-generating capacity at a given joint angle, the force-length properties of the MTUs are the primary drivers of varying strength capabilities at different joint angles. *In vitro*, maximally stimulated muscle fibers attain their greatest active isometric force when actin-myosin overlap is maximized. If muscle fiber length is shorter or longer than this optimum, active force decreases (Rassier et al., 1999). During an isometric voluntary contraction *in vivo* (fixed-end contraction) sarcomere length decreases and the magnitude of shortening depends on the contraction force and tendon stiffness. Changing the joint angle leads to both alteration of the actin-myosin overlap and tendon stiffness which influence the voluntary torque-generating capacity (Rassier et al., 1999; MacIntosh, 2017). The same applies to submaximal electrically induced contractions. However, in this case the torque-joint angle relationship shifts to the right compared the MVCs. This means, for example, that the peak twitch torque is produced at a larger joint angle. The rightward shift of the torque-joint angle relationship is partly abolished by activity-induced potentiation, i.e., potentiation of the electrically induced twitch torque following a short MVC. Although activity-induced potentiation alters the torque-joint angle relationship, the lowest submaximal electrically induced unpotentiated and potentiated twitch torques are usually observed at the shortest MTU length (Hansen et al., 2003; MacIntosh, 2017).

Although the length dependence of twitch properties and their activity-induced potentiation are known for several muscle groups surrounding different joints (Bigland-Ritchie et al., 1992; Rassier, 2000; Hansen et al., 2003), these relationships are not established for the wrist flexors innervated by the median nerve.

Insights into the neural and muscular properties of the wrist flexors at different wrist joint angles could help to understand normal wrist function, which is a first step in determining and treating musculoskeletal disorders. Therefore, we investigated neuromuscular properties of the human wrist flexors innervated by the median nerve as a function of the wrist joint angle. We analyzed isometric voluntary and electrically evoked contractions of the wrist flexors at three randomly assigned wrist joint angles (extension: −30°, neutral: 0°, flexion: 30°) to quantify the joint angle dependence of (i) voluntary activation, (ii) unpotentiated twitch torque properties, and (iii) potentiated twitch torque properties.

Based on the literature presented above, we hypothesized that voluntary activation and unpotentiated twitch torques decrease the shorter the MTU. In addition, we conjectured that activity-induced potentiation partly abolishes angle-dependent differences in unpotentiated twitch torques.

## Materials and Methods

### Participants

Two recently published studies investigating the effect of different knee joint angles on voluntary activation of the knee extensors did not provide sufficient information to allow for effect size calculation for the present study (Doguet et al., 2017b; Lanza et al., 2017). Therefore, a sample size calculation was performed assuming a medium effect size (*f* = 0.25) for the change in voluntary activation between joint angles, with an α level of 0.05, a power (1–β) of 0.8, and a correlation among repeated measures of 0.7 (G^*^Power, version 3.1.9.2, University of Kiel, Kiel, Germany). Sample size calculation indicated that 17 participants would be required. To account for potential drop outs, 18 recreationally active, right-handed males (age: 26.8 ± 3.8 years, height: 183.7 ± 8.0 cm, body mass: 84.8 ± 7.7 kg, exercise involving the upper body per week: 3.1 ± 3.9 h) without known neurological and/or orthopedic disorders of the left arm were recruited. Handedness of the participants was determined by the Edinburgh handedness inventory (Oldfield, 1971). Participants were informed about the experimental procedures as well as the discomfort and possible risks associated with the experiment before giving their written informed consent. In addition, the participants were asked to refrain from strenuous exercise involving the upper body as well as alcohol and caffeine consumption 48 h prior to the experiment. The study was conducted according to the declaration of Helsinki and was approved by the local ethics committee.

### Experimental Protocol

**Figure 1:**
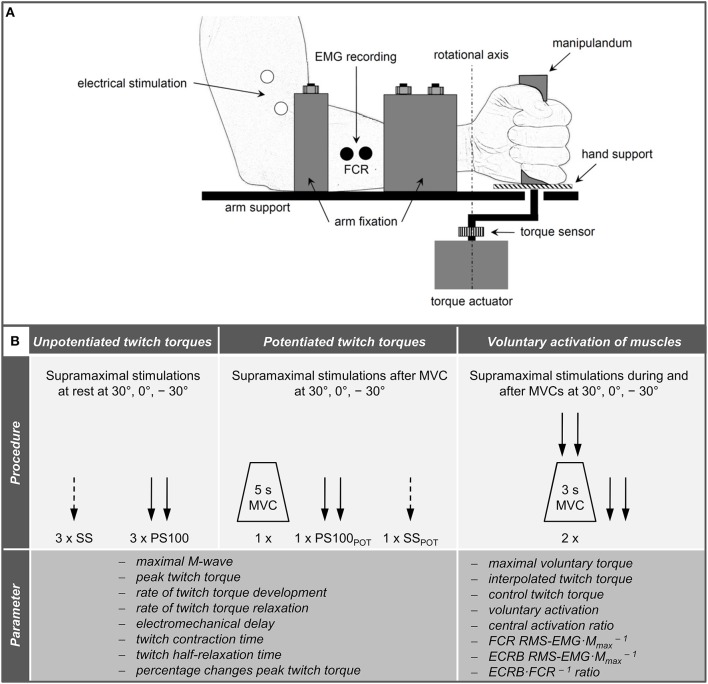
Schematic representation of the experimental setup **(A)** and chronology of the procedures carried out during neuromuscular testing and the extracted parameters **(B)**. Please note that the hand fixation is not displayed and the order of the wrist joint angles was randomized. ECRB, extensor carpi radialis brevis; FCR, flexor carpi radialis; MVC, maximal voluntary contraction; PS100, unpotentiated twitch torques evoked by paired electrical stimuli at 100 Hz [inter-stimulus interval (ISI) 10 ms]; PS100_POT_, potentiated twitch torques evoked by paired electrical stimuli at 100 Hz [ISI 10 ms] RMS·M^−1^, normalized root mean square of the EMG signal; SS, unpotentiated twitch torques evoked by single electrical stimuli; SS_POT_, potentiated twitch torques evoked by single electrical stimuli; −30°, extension: long wrist flexor muscle length; 0°, neutral: intermediate wrist flexor muscle length; 30°, flexion: short wrist flexor muscle length.

The measurements were conducted on the left forearm to minimize confounding effects of occupational and dominant hand use in our right-handed participants. During neuromuscular testing, participants were comfortably seated in a standardized position on a chair belonging to a custom-built dynamometer which was designed to measure wrist flexion and extension torques (Figure 1A). The experimental protocol involved two sessions on separate days. During the first session, participants were thoroughly familiarized with the neuromuscular testing procedures that consisted of electrical stimulation of the median and radial nerve at rest as well as during isometric MVCs of the wrist flexors at three different wrist joint angles, i.e., extension (−30°), neutral (0°), and flexion (30°), respectively. During the second session, participants' neuromuscular function of the wrist flexors innervated by the median nerve was measured at rest and during isometric MVCs at the three randomly assigned wrist joint angles (−30°, 0°, 30°). First of all, unpotentiated twitch torques of the wrist flexors in response to single (SS) and paired [PS100, inter-stimulus interval (ISI) 10 ms, 100 Hz] supramaximal electrical stimuli were recorded. Afterwards, the participants performed a MVC of the wrist flexors for 5 s and potentiated twitch torques were evoked by paired (PS100_POT_) and single (SS_POT_) supramaximal electrical stimuli 5 and 10 s following the MVC, respectively. Then, voluntary activation of the wrist flexors innervated by the median nerve was assessed using the interpolated twitch technique (Allen et al., 1995). The order of the wrist joint angles during neuromuscular testing involving MVCs was the same as during neuromuscular testing at rest (Figure 1B).

### Torque Recordings

**Figure 2:**
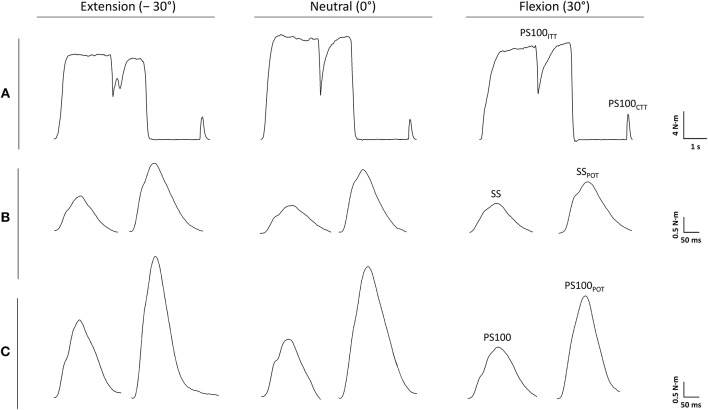
Torque signals produced by voluntary **(A)** and electrically evoked contractions **(B,C)** of one representative participant. PS100, unpotentiated twitch torque evoked by paired electrical stimuli at 100 Hz [inter-stimulus interval (ISI) 10 ms]; PS100_CTT_, control twitch torque evoked by paired electrical stimuli at 100 Hz (ISI 10 ms); PS100_ITT_, interpolated twitch torque evoked by paired electrical stimuli at 100 Hz (ISI 10 ms); PS100_POT_, potentiated twitch torque evoked by paired electrical stimuli at 100 Hz (ISI 10 ms); SS, unpotentiated twitch torque evoked by a single electrical stimulus; SS_POT_, potentiated twitch torque evoked by a single electrical stimulus. −30° extension: long wrist flexor muscle length; 0°, neutral: intermediate wrist flexor muscle length; 30°, flexion: short wrist flexor muscle length.

A custom-built dynamometer was used to measure electrically evoked and voluntary torques. The participants' left hands were fixed to a manipulandum with a Velcro strap and the fingers were flexed. The manipulandum consists of a rotatable vertical handle linked to the lever arm of a torque motor (145ST4M, ALXION, France). Furthermore, the left forearms of the participants were secured with a fixation unit covering its entire length except the locations where the EMG electrodes were placed. The distal part of the fixation unit was locked 3 cm proximal to the styloid process of the ulna. Figure 1A shows a schematic representation of the experimental setup. A constant position of the subjects' head was guaranteed using an adjustable chin rest. The elbow flexion and shoulder abduction angles were 107 ± 6° and 42 ± 8°, respectively. In this study, the neutral (0°) wrist joint angle was set when the longitudinal axis of the forearm was in line with the center of the manipulandum (Jung and Hallbeck, 2002; Morse et al., 2006). The rotation axis of the dynamometer was in line with the wrist joint rotation axis. During isometric strength testing, participants were instructed to perform isometric wrist flexions as hard as possible against the manipulandum of the dynamometer with at least 2 min rest in between. Strong verbal encouragement was given by the investigator. Visual feedback of the torque-time curve was provided on a digital oscilloscope (HM1507-3, HAMEG® Instruments, Germany). The torque signals were acquired using a torque sensor (9049; Kistler, Germany). Signals were digitized with a sampling frequency of 3 kHz using an analog-to-digital converter (DAQ CARD™-6024E, National Instruments, USA). Data were saved on a hard drive for later analysis using a custom-built LABVIEW based program (Imago, Pfitec, Germany). An example of the torque signals produced by isometric voluntary and electrically evoked contractions of one representative participant is presented in Figure 2.

### EMG Recordings

Myoelectric signals from the flexor carpi radialis muscle (FCR) and extensor carpi radialis brevis muscle (ECRB) were recorded using surface electrodes (EMG Ambu® Blue Sensor N). The self-adhesive electrodes were attached to the shaved, abraded, and cleaned skin over the muscle bellies and in line with the presumed direction of the muscle fibers (center-to-center distance of 2 cm). A reference electrode was attached to the ipsilateral olecranon. The resistance between electrodes was kept below 5 kΩ. EMG signals were amplified (2,500 ×), band-pass filtered (10–450 Hz) and digitized with a sampling frequency of 3 kHz using an analog-to-digital converter (DAQ CARD™-6024E, National Instruments, USA). Data were saved on a hard drive for later analysis using a custom-built LABVIEW based program (Imago, Pfitec, Germany).

### Electrical Nerve Stimulation

Single electrical stimuli were delivered to the radial nerve via self-adhesive surface electrodes (15 × 20 mm, Spes Medica, Italy) with the cathode over the spiral groove and the anode placed over the biceps brachii muscle (Burke, 2016). The median nerve was electrically stimulated via self-adhesive surface electrodes (15 × 20 mm, Spes Medica, Italy) near the cubital fossa with the cathode proximal to the anode (Lee and Carroll, 2005). Stimulation electrode positioning was individually optimized to ensure that electrical stimulation of the median nerve did not activate the antagonist significantly (ECRB). A constant-current stimulator (DS7A, Digitimer, United Kingdom) was used to deliver square-wave pulses of 1 ms duration with a maximal voltage of 400 V. Individual electrical stimulation intensity was progressively increased until the maximum compound muscle action potential (M_max_) of FCR and ECRB as well as a plateau in wrist flexor and extensor twitch torque, respectively, was reached. This procedure was performed at three wrist joint angles (−30°, 0°, 30°). During the subsequent neuromuscular testing procedures, a supramaximal electrical stimulation intensity (140%) was used. The spread of the supramaximal electrical stimuli to the ECRB was continuously monitored by M-wave recording and was similar for each wrist joint angle [*F*_(2, 34)_ = 0.694, *P* = 0.506, $\eta_{\text{p}}^{2}$ = 0.039]. Contractile properties of the wrist flexors were assessed using unpotentiated (SS and PS100) and potentiated (SS_POT_ and PS100_POT_) twitch torques evoked by single and paired (ISI 10 ms, 100 Hz) electrical stimuli, respectively. This technique has been used previously to analyze the effect of immobilization on contractile function of the wrist flexors innervated by the median nerve (Lundbye-Jensen and Nielsen, 2008). In order to determine the level of voluntary activation of the wrist flexors innervated by the median nerve during isometric MVCs at the three wrist joint angles (−30°, 0°, 30°), the interpolated twitch technique was applied. For this purpose, supramaximal electrical paired stimuli were delivered to the median nerve 2 s after torque onset (during the plateau phase) and 2 s after MVC. This technique was previously used to determine voluntary activation of the wrist flexors innervated by the median nerve in young and old adults (Clark et al., 2015) as well as central factors of wrist flexor muscle fatigue (Hartley et al., 2016).

### Data Analyses

Unpotentiated (SS and PS100) and potentiated (SS_POT_ and PS100_POT_) twitch torques were analyzed to quantify peak twitch torque, rate of twitch torque development (peak twitch torque × twitch contraction time^−1^), rate of twitch torque relaxation (0.5 × peak twitch torque × twitch half-relaxation time^−1^), electromechanical delay (time interval between EMG and torque onset), twitch contraction time (time interval between torque onset and peak twitch torque), and twitch half-relaxation time (time interval between peak twitch torque and 50% decline in peak twitch torque), respectively. In order to investigate the angle-specific magnitude of activity-induced peak twitch torque potentiation, the percentage changes between peak twitch torque SS and SS_POT_ as well as between peak twitch torque PS100 and PS100_POT_ were calculated. In addition, the contribution of a second supramaximal electrical stimulus to the unpotentiated and potentiated peak twitch torque production was analyzed, i.e., the percentage changes between peak twitch torque SS and PS100 as well as between peak twitch torque SS_POT_ and PS100_POT_ were computed.

Isometric MVTs were determined on the basis of the torque-time curves, i.e., the highest torque value immediately before the application of the electrical stimuli.

Calculation of percentage voluntary activation was done with the corrected formula that takes the torque value immediately before the occurrence of the interpolated twitch torque (T_b_) into account: voluntary activation = (1 – interpolated twitch torque × (T_b_ × MVT^−1^) × control twitch torque^−1^) (Strojnik and Komi, 1998). This corrected formula counteracts the problem that the superimposed stimuli are not always applied at the instant of MVT. It has recently been shown that voluntary activation of the knee extensors during isometric MVCs can be reliably assessed using this formula (Behrens et al., 2017). In addition, the central activation ratio (Kent-Braun and Le Blanc, 1996) was calculated as follows: central activation ratio = MVT × (MVT + interpolated twitch torque)^−1^ × 100. The central activation ratio has been shown to overestimate the neural drive to muscles in comparison with the measurement of voluntary activation incorporating a control twitch torque (Place et al., 2007). However, this technique excludes the bias introduced by a different muscle length which is present when evoking the interpolated twitch torque during MVC compared to evoking the control twitch torque at rest. This is of particular importance when the neural drive is assessed at a short muscle length (Arampatzis et al., 2007).

Agonistic and antagonistic muscle activity during MVCs of the wrist flexors was expressed as root mean square of the EMG signal which was normalized to M_max_ peak-to-peak amplitudes evoked by single stimuli (RMS·M^−1^). In addition, the co-activation ratio during MVC of the wrist flexors was computed by dividing ECRB RMS·M^−1^ values by FCR RMS·M^−1^ values (ECRB·FCR^−1^ ratio).

### Statistical Analyses

Data were screened for normal distribution using the Shapiro-Wilk test. One-way repeated measures Analyses of Variance (ANOVAs) with wrist joint angle (−30°, 0°, 30°) as within-subject variable were conducted. In case of statistical significant differences in the parameters between the wrist joint angles, *post-hoc* analyses were carried out with Bonferroni corrections. Effect sizes were expressed as partial eta-squared ($\eta_{\text{p}}^{2}$). The level of significance was set at *P* ≤ 0.050. Data were analyzed using the SPSS statistical package 22.0 (SPSS Inc., USA).

## Results

The data of all participants were collected successfully. Significant angle-specific differences were observed for several parameters. The following listing reports the statistical outcomes for the ANOVAs. Absolute values (means ± standard deviations) and results of *post-hoc* comparisons are provided in Table 1.

**Table 1:** Neuromuscular function of the wrist flexors depending on the wrist joint angle.

	**Wrist joint angle**			***P*****-values**		
	**Extension (– 30°)**	**Neutral (0°)**	**Flexion (30°)**	**−30° vs. 0°**	**−30° vs. 30°**	**30° vs. 0°**
Maximal voluntary torque (N·m)	16.08 ± 2.78	19.17 ± 3.12	18.46 ± 2.46	<0.001^**^	0.001^**^	0.552
Voluntary activation (%)	98.11 ± 2.65	97.40 ± 3.52	96.06 ± 4.05	0.742	0.005^**^	0.055
Central activation ratio (%)	99.21 ± 1.05	99.20 ± 1.01	98.89 ± 1.06	n.a.	n.a.	n.a.
Interpolated twitch torque (N·m)	0.118 ± 0.154	0.149 ± 0.186	0.200 ± 0.191	1	0.039^*^	0.186
Control twitch torque (N·m)	6.02 ± 0.93	5.68 ± 0.85	5.17 ± 0.87	0.192	0.002^**^	0.017^*^
**RMS·M**^−1^						
Flexor carpi radialis (FCR)	0.088 ± 0.023	0.087 ± 0.030	0.084 ± 0.030	n.a.	n.a.	n.a.
Extensor carpi radialis brevis (ECRB)	0.042 ± 0.018	0.041 ± 0.016	0.046 ± 0.015	n.a.	n.a.	n.a.
ECRB·FCR^−1^ ratio	0.50 ± 0.24	0.52 ± 0.23	0.62 ± 0.30	1	0.021^*^	0.174
**M_max_ (mV)**						
FCR	9.00 ± 3.03	9.29 ± 2.95	10.63 ± 3.77	1	0.009^**^	0.033^*^
ECRB	6.98 ± 2.53	6.90 ± 2.05	6.47 ± 2.03	n.a.	n.a.	n.a.
**Peak twitch torque (N·m)**						
SS	1.58 ± 0.34	1.25 ± 0.28	0.98 ± 0.24	<0.001^**^	<0.001^**^	<0.001^**^
PS100	4.01 ± 0.65	3.44 ± 0.66	2.90 ± 0.51	<0.001^**^	<0.001^**^	<0.001^**^
SS_POT_	2.99 ± 0.38	2.69 ± 0.44	2.29 ± 0.36	0.006^**^	<0.001^**^	<0.001^**^
PS100_POT_	6.29 ± 0.99	5.93 ± 0.92	5.40 ± 0.95	0.31	<0.001^**^	0.06
**Rate of twitch torque development (N·m·s**^–1^**)**						
SS	23.16 ± 4.27	18.55 ± 3.46	15.63 ± 4.00	<0.001^**^	<0.001^**^	0.032^*^
PS100	52.32 ± 9.22	47.10 ± 8.76	40.40 ± 7.12	0.009^**^	<0.001^**^	0.001^**^
SS_POT_	53.42 ± 8.91	50.30 ± 7.84	45.01 ± 7.11	0.46	0.001^**^	0.067
PS100_POT_	86.32 ± 14.74	85.98 ± 12.68	83.27 ± 15.70	n.a.	n.a.	n.a.
**Rate of twitch torque relaxation (N·m·s**^–1^**)**						
SS	12.77 ± 3.05	11.09 ± 2.94	9.65 ± 2.87	0.009^**^	0.001^**^	0.016^*^
PS100	31.60 ± 5.44	30.68 ± 5.35	29.30 ± 5.93	n.a.	n.a.	n.a.
SS_POT_	30.01 ± 4.15	30.37 ± 4.05	27.91 ± 4.64	n.a.	n.a.	n.a.
PS100_POT_	52.15 ± 9.71	56.36 ± 6.86	57.76 ± 7.94	0.114	0.038^*^	1
**Electromechanical delay (ms)**						
SS	9.04 ± 0.82	9.80 ± 1.03	11.61 ± 2.65	0.009^**^	0.004^**^	0.017^*^
PS100	9.11 ± 0.78	9.87 ± 1.07	11.70 ± 2.55	0.004^**^	0.002^**^	0.008^**^
SS_POT_	8.56 ± 0.96	8.94 ± 0.85	10.72 ± 1.63	0.556	<0.001^**^	<0.001^**^
PS100_POT_	8.22 ± 1.18	9.22 ± 1.03	10.67 ± 1.70	0.073	<0.001^**^	0.001^**^
**Twitch contraction time (ms)**						
SS	68.24 ± 5.27	67.31 ± 5.29	63.41 ± 8.49	1	0.029^*^	0.211
PS100	77.09 ± 5.85	73.02 ± 3.98	71.94 ± 5.61	<0.001^**^	0.002^**^	0.757
SS_POT_	56.56 ± 6.18	53.61 ± 4.98	51.78 ± 8.97	0.173	0.074	0.701
PS100_POT_	73.06 ± 4.01	69.06 ± 5.15	65.44 ± 6.52	0.003^**^	<0.001^**^	0.036^*^
**Twitch half-relaxation time (ms)**						
SS	63.17 ± 9.92	57.63 ± 7.67	51.97 ± 6.94	0.019^*^	0.002^**^	0.010^**^
PS100	63.96 ± 7.64	56.09 ± 5.52	50.09 ± 7.08	0.001^**^	<0.001^**^	0.001^**^
SS_POT_	50.33 ± 7.10	44.78 ± 8.50	41.67 ± 6.19	<0.001^**^	<0.001^**^	0.406
PS100_POT_	61.00 ± 7.13	52.72 ± 6.23	46.72 ± 5.01	<0.001^**^	<0.001^**^	<0.001^**^
**Percentage changes peak twitch torque**						
SS vs. SS_POT_	92.84 ± 27.71	119.59 ± 34.52	142.88 ± 49.17	<0.001^**^	<0.001^**^	0.111
PS100 vs. PS100_POT_	57.82 ± 15.44	75.75 ± 26.12	88.74 ± 28.77	0.010^**^	0.002^**^	0.189
SS vs. PS100	158.89 ± 47.04	180.52 ± 54.37	205.49 ± 60.15	0.013^*^	<0.001^**^	0.002^**^
SS_POT_ vs. PS100_POT_	111.23 ± 26.52	122.66 ± 28.02	136.56 ± 31.61	0.074	<0.001^**^	0.063

*Please note that post-hoc comparisons are only available if the ANOVA with repeated measures indicated a statically significant difference (P-values in bold are significant*,

* *P ≤ 0.050*,

** *P ≤ 0.010). n. a., not applicable because the ANOVA did not indicate a main effect of angle; RMS·M^−1^, root mean square of the EMG signal normalized to the maximal M-wave (M_max_); SS, unpotentiated peak twitch torque evoked by a single stimulus; PS100, unpotentiated peak twitch torque evoked by paired stimuli at 100 Hz [inter-stimulus interval (ISI) 10 ms]; SS_POT_, potentiated peak twitch torque evoked by a single stimulus; PS100_POT_, potentiated peak twitch torque evoked by paired stimuli at 100 Hz (ISI 10 ms). Values are expressed as means ± standard deviations*.

### Maximal Voluntary Torque, Voluntary Activation, Central Activation Ratio, and EMG Parameters

The statistical analysis yielded a significant angle-dependent difference in isometric MVT [*F*_(2, 34)_ = 25.120, *P* < 0.001, $\eta_{\text{p}}^{2}$ = 0.596] with the lowest value in extension (−30°). Voluntary activation of the wrist flexors declined with decreasing MTU length [*F*_(2, 34)_ = 7.062, *P* = 0.003, $\eta_{\text{p}}^{2}$ = 0.293] due to angle-specific alterations of the interpolated twitch torque [*F*_(2, 34)_ = 3.674, *P* = 0.036, $\eta_{\text{p}}^{2}$ = 0.178] and control twitch torque [*F*_(2, 34)_ = 11.670, *P* < 0.001, $\eta_{\text{p}}^{2}$ = 0.407]. The central activation ratio pointed to the same direction although not statistically significant [*F*_(2, 34)_ = 2.364, *P* = 0.109, $\eta_{\text{p}}^{2}$ = 0.122].

**Figure 3:**
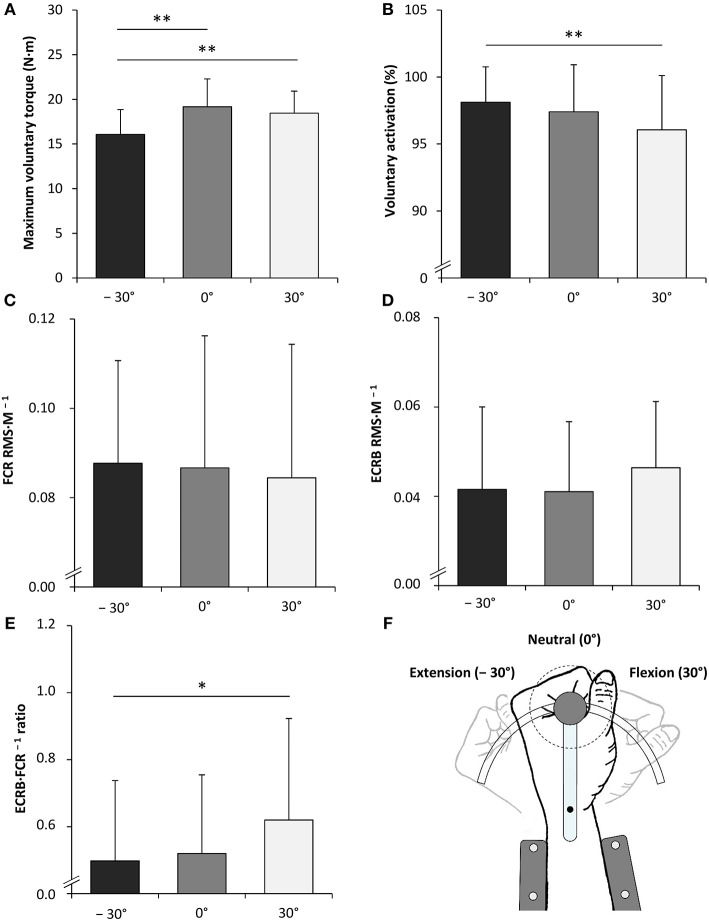
Maximal voluntary torque of the wrist flexors **(A)**, voluntary activation of the wrist flexors **(B)**, root mean square of the EMG signal normalized to the maximal M-wave (RMS·M^−1^) of the flexor carpi radialis (FCR) during MVC of the wrist flexors **(C)**, RMS·M^−1^ of the extensor carpi radialis brevis (ECRB) during MVC of the wrist flexors **(D)**, the antagonist·agonist^−1^ EMG ratio (ECRB·FCR^−1^ ratio) during MVC of the wrist flexors **(E)** and the schematic representation of the experimental setup **(F)**. −30°, extension: long wrist flexor muscle length; 0°, neutral: intermediate wrist flexor muscle length; 30°, flexion: short wrist flexor muscle length. *Denotes a significant difference between joint angles (*P ≤ 0.050, **P ≤ 0.010). Values are expressed as means ± standard deviations.

Angle-dependent differences were also observed for some EMG data. The statistical analysis revealed a significantly increased FCR M_max_ amplitude with the highest value in flexion (30°) [*F*_(2, 34)_ = 7.762, *P* = 0.002, $\eta_{\text{p}}^{2}$ = 0.313]. The same was true for the ECRB·FCR^−1^ ratio [*F*_(2, 34)_ = 4.801, *P* = 0.015, $\eta_{\text{p}}^{2}$ = 0.220] (Figure 3, Table 1).

### Unpotentiated and Potentiated Twitch Torque Properties

**Figure 4:**
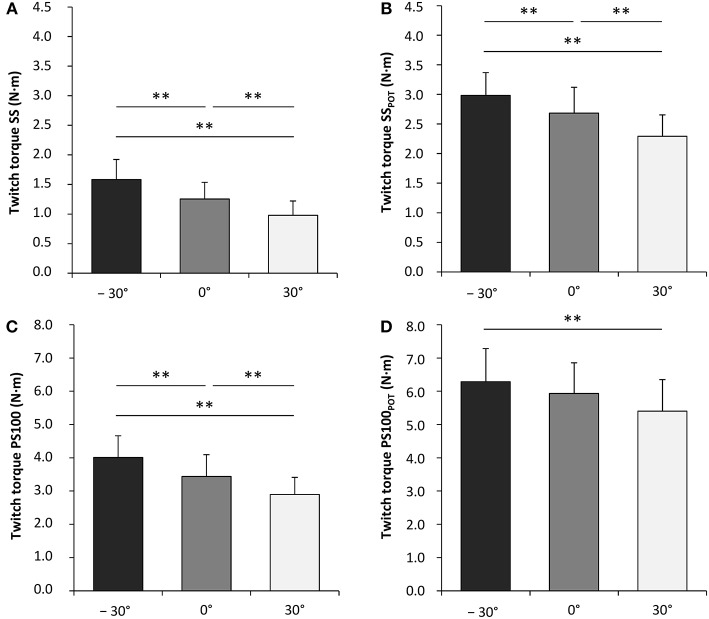
Unpotentiated peak twitch torques evoked by single electrical stimuli [ss] **(A)**, potentiated peak twitch torques evoked by single electrical stimuli [SS_POT_] **(B)**, unpotentiated peak twitch torques evoked by paired electrical stimuli at 100 Hz [inter-stimulus interval (ISI) 10 ms, PS100] **(C)** and potentiated peak twitch torques evoked by paired electrical stimuli at 100 Hz (ISI 10 ms, PS100_POT_) **(D)**. −30°, extension: long wrist flexor muscle length; 0°, neutral: intermediate wrist flexor muscle length; 30°, flexion: short wrist flexor muscle length. *Denotes a significant difference between joint angles (**P ≤ 0.010). Values are expressed as means ± standard deviations.

Contractile properties of the wrist flexors were significantly modulated as a function of the wrist joint angle. The peak twitch torque increased from flexion (30°) over neutral (0°) to extension (−30°) for SS [*F*_(2, 34)_ = 65.599, *P* < 0.001, $\eta_{\text{p}}^{2}$ = 0.794], PS100 [*F*_(2, 34)_ = 45.058, *P* < 0.001, $\eta_{\text{p}}^{2}$ = 0.726], SS_POT_ [*F*_(2, 34)_ = 40.034, *P* < 0.001, $\eta_{\text{p}}^{2}$ = 0.702], and PS100_POT_ [*F*_(2, 34)_ = 10.061, *P* < 0.001, $\eta_{\text{p}}^{2}$ = 0.372] (Figure 4, Table 1). The same was true for rate of twitch torque development for SS [*F*_(2, 34)_ = 35.196, *P* < 0.001, $\eta_{\text{p}}^{2}$ = 0.674], PS100 [*F*_(2, 34)_ = 25.674, *P* < 0.001, $\eta_{\text{p}}^{2}$ = 0.602], and SS_POT_ [*F*_(2, 34)_ = 8.856, *P* = 0.001, $\eta_{\text{p}}^{2}$ = 0.343]. Rate of twitch torque relaxation was significantly altered due to changes in the wrist joint angle only for SS [*F*_(2, 34)_ = 16.850, *P* < 0.001, $\eta_{\text{p}}^{2}$ = 0.498] and PS100_POT_ [*F*_(2, 34)_ = 4.612, *P* = 0.017, $\eta_{\text{p}}^{2}$ = 0.213]. Angle-specific changes in electromechanical delay were observed for SS [*F*_(2, 34)_ = 12.646, *P* < 0.001, $\eta_{\text{p}}^{2}$ = 0.427], PS100 [*F*_(2, 34)_ = 15.200, *P* < 0.001, $\eta_{\text{p}}^{2}$ = 0.472], SS_POT_ [*F*_(2, 34)_ = 21.140, *P* < 0.001, $\eta_{\text{p}}^{2}$ = 0.554], and PS100_POT_ [*F*_(2, 34)_ = 19.138, *P* < 0.001, $\eta_{\text{p}}^{2}$ = 0.530] with the lowest value at a wrist joint angle of −30°. Twitch contraction time increased from flexion (30°) over neutral (0°) to extension (−30°) for SS [*F*_(2, 34)_ = 5.053, *P* = 0.012, $\eta_{\text{p}}^{2}$ = 0.229], PS100 [*F*_(2, 34)_ = 15.068, *P* < 0.001, $\eta_{\text{p}}^{2}$ = 0.470], SS_POT_ [*F*_(2, 34)_ = 4.331, *P* = 0.021, $\eta_{\text{p}}^{2}$ = 0.203], and PS100_POT_ [*F*_(2, 34)_ = 17.759, *P* < 0.001, $\eta_{\text{p}}^{2}$ = 0.511]. The same applied to the twitch half-relaxation time for SS [*F*_(2, 34)_ = 13.941, *P* < 0.001, $\eta_{\text{p}}^{2}$ = 0.451], PS100 [*F*_(2, 34)_ = 30.413, *P* < 0.001, $\eta_{\text{p}}^{2}$ = 0.641], SS_POT_ [*F*_(2, 34)_ = 14.235, *P* < 0.001, $\eta_{\text{p}}^{2}$ = 0.456], and PS100_POT_ [*F*_(2, 34)_ = 49.014, *P* < 0.001, $\eta_{\text{p}}^{2}$ = 0.742; Table 1].

**Figure 5:**
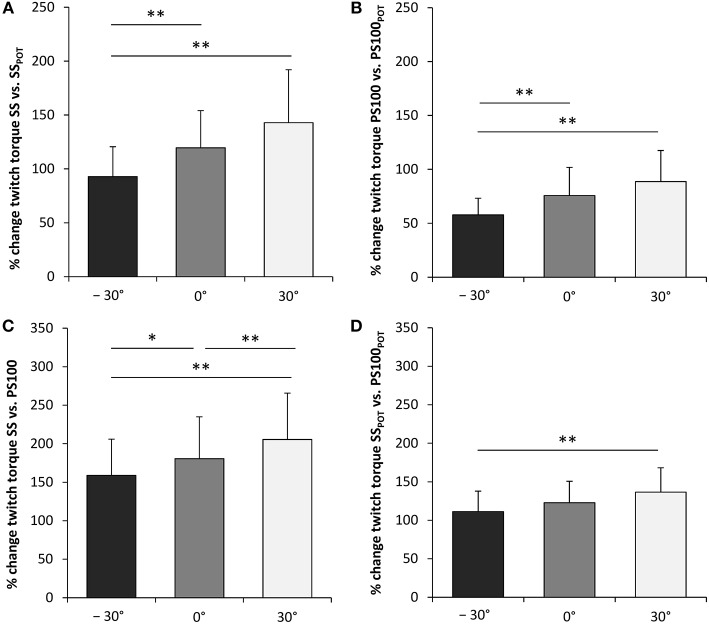
Activity-induced potentiation (%) of peak twitch torques evoked by single **(A)** and paired electrical stimuli **(B)**. Percentage contribution of a second electrical stimulus to the unpotentiated **(C)** and the potentiated peak twitch torque production **(D)**. SS, unpotentiated peak twitch torque evoked by a single stimulus; PS100, unpotentiated peak twitch torque evoked by paired stimuli at 100Hz [inter-stimulus interval (ISI) 10 ms]; SS_POT_, potentiated peak twitch torque evoked by a single stimulus; PS100_POT_, potentiated peak twitch torque evoked by paired stimuli at 100Hz (ISI 10 ms); −30°, extension: long wrist flexor muscle length; 0°, neutral: intermediate wrist flexor muscle length; 30°, flexion: short wrist flexor muscle length. *Denotes a significant difference between joint angles (*P ≤ 0.050, **P ≤ 0.010). Values are expressed as means ± standard deviations.

Wrist joint angle had a significant effect on percentage changes between peak twitch torque SS and SS_POT_ [*F*_(2, 34)_ = 15.826, *P* < 0.001, $\eta_{\text{p}}^{2}$ = 0.482] as well as between peak twitch torque PS100 and PS100_POT_ [*F*_(2, 34)_ = 11.807, *P* < 0.001, $\eta_{\text{p}}^{2}$ = 0.410], i.e., highest activity-induced potentiation in flexion (30°). The same applied for the contribution of a second supramaximal electrical stimulus to the unpotentiated and potentiated peak twitch torque production, i.e., the angle-dependent percentage changes between peak twitch torque SS and PS100 [*F*_(2, 34)_ = 21.771, *P* < 0.001, $\eta_{\text{p}}^{2}$ = 0.562] as well as between peak twitch torque SS_POT_ and PS100_POT_ [*F*_(2, 34)_ = 13.581, *P* < 0.001, $\eta_{\text{p}}^{2}$ = 0.444] (Figure 5, Table 1).

## Discussion

The present study investigated, for the first time, neuromuscular properties of the human wrist flexors innervated by the median nerve at three different wrist joint angles (−30°, 0°, 30°). The MVT was lower in extension (−30°) compared to neutral (0°) and flexion (30°). Although voluntary activation was generally high, data indicate that voluntary activation of the wrist flexors was lower with the wrist joint in flexion (30°) compared with the wrist joint in extension (−30°), i.e., short vs. long length of the MTU, respectively. This was accompanied by an increased antagonist·agonist^−1^ EMG ratio during MVC of the wrist flexors with the wrist joint in flexion (30°). We further found that peak twitch torque, rate of twitch torque development, and twitch half-relaxation time increased, whereas electromechanical delay decreased from flexion (30°) to extension (−30°) for the unpotentiated twitch torques (SS and PS100). Activity-induced potentiation partly abolished these differences and was highest in short compared to long muscles.

In the present study, MVT of the wrist flexors was lowest with the wrist joint in extension (−30°) compared with the wrist joint in the flexed (30°) and neutral (0°) position. This result is in accordance with the outcome of studies that have analyzed strength capabilities of the wrist flexors at different wrist joint angles (Delp et al., 1996; Gonzalez et al., 1997). The MVT at different joint angles is not only a result of the interplay between neural and muscular contractile factors, but is also determined by the variable angle-dependent moment arms of the muscle force vectors as well as the pennation angles of muscle fibers (Herbert and Gandevia, 1995; Gonzalez et al., 1997).

In agreement with the observations of previously published experiments investigating the joint angle dependence of neural drive to the knee extensors (Becker and Awiszus, 2001; Kubo et al., 2004; Doguet et al., 2017b; Lanza et al., 2017), we found that voluntary activation of shorter wrist flexors was lower compared to that of longer wrist flexors. However, in this regard it has to be considered that the assessment of voluntary activation using the interpolated twitch technique requires a control twitch that is elicited by stimulating the resting muscle via its supplying peripheral nerve. This approach introduces the problem that MTU length during MVC and at rest differs which affects the size of the control twitch. If the length of the MTU decreases, its compliance increases, which adversely influences the peak twitch torque of the control twitch (Arampatzis et al., 2007). Therefore, the central activation ratio, which does not require a control twitch, was calculated as well. Although the ANOVA did not indicate a significant difference in the central activation ratio between the three wrist joint angles [*F*_(2, 34)_ = 2.364, *P* = 0.109, $\eta_{\text{p}}^{2}$ = 0.122], a tendency toward lower activation at short muscle length was demonstrable (Table 1). When directly comparing the values recorded with the wrist joint in flexion (30°) and extension (−30°) using a paired *t*-test, a strong tendency for a lower central activation ratio at short compared to long MTU length with a nearly medium effect size was observable (*P* = 0.061, Cohen's d = 0.481). This secondary data analysis supports the notion that voluntary activation of short muscles is lower compared to that of long muscles (Becker and Awiszus, 2001; Kubo et al., 2004; Doguet et al., 2017b; Lanza et al., 2017) and that this relationship could exist for the wrist flexors as well.

### Joint Angle Dependence of Voluntary Activation and Potential Contributing Mechanisms

The discussed mechanisms driving the joint angle dependence of voluntary activation involve excitatory and inhibitory feedback originating from muscle spindle afferents and other mechanoreceptors (i.e., ligament receptors, joint receptors, and Golgi tendon organs) (Becker and Awiszus, 2001; Kubo et al., 2004; Doguet et al., 2017b; Lanza et al., 2017).

#### Feedback From Muscle Spindles

It has been proposed that muscle spindle discharge rates are higher in long compared to short muscles leading to an increased excitation of the α-motoneuron pool and, in turn, an elevated voluntary activation of muscles (Becker and Awiszus, 2001; Kubo et al., 2004). However, results of experiments analyzing the discharge rates of muscle spindle primary afferents of the finger flexors as well as H-reflexes of the wrist and plantar flexors during static voluntary contractions at different MTU lengths do not support this assumption (Jahnke and Struppler, 1990; Chen et al., 2006; Papaiordanidou et al., 2016). These data suggest that the input from muscle spindles, the excitability of spinal α-motoneurons via Ia afferents, and the effectiveness of Ia synaptic transmission are not significantly different during MVCs at different MTU lengths. In contrast to that, it has been shown recently that corticospinal excitability of the knee extensors during isometric MVCs was higher at short compared to long MTU length (Doguet et al., 2017a). Given that motor evoked potential amplitudes can be altered by changes in excitability at the cortical and/or spinal level, the combined measurement of motor evoked potentials and thoracic/cervicomedullary motor evoked potentials might clarify cortical and spinal contributions to the angle dependence of motor evoked potentials (Behrens, 2017). Furthermore, the measurement of intracortical and interhemispheric inhibition or facilitation could provide additional information in this regard.

#### Non-reciprocal Group I Inhibition

A contribution of nonreciprocal group I inhibition, associated with Golgi tendon organs, to the decreased voluntary activation at short compared to long MTU length seems unlikely because non-reciprocal group I inhibition is suppressed during voluntary contractions (Pierrot-Deseilligny and Burke, 2012). Nonetheless, there are several other inputs to interneurons mediating non-reciprocal group I inhibition, e.g., from descending tracts, joint afferents, ligament afferents, and cutaneous afferents that have the potential to alter the extent of inhibition.

#### Feedback From Joint and Ligament Afferents

Activation of mechanoreceptors in ligaments has been shown to alter muscle activity directly in order to assist joint stabilization and co-contraction (Solomonow, 2006). The dorsal ligaments of the wrist possess a larger number of mechanoreceptors compared to the palmar ligaments and they are probably under tension during wrist flexion (Hagert, 2010). It is conceivable that the neural drive to the wrist flexor and extensor muscles during wrist flexion (30°) was modulated by input from joint and ligament afferents which project to spinal and supraspinal centers.

#### Persistent Inward Currents

In addition, has been recently shown that persistent inward currents of ankle extensor motoneurons in cats are modulated by ankle joint rotations. The amplitude of persistent inward currents was highest when the ankle was flexed (long MTU length) and was lowest when the ankle was extended (short MTU length) (Hyngstrom et al., 2007). Currently, it is unclear if and to what extent this mechanism plays a role during isometric MVCs.

#### Recurrent Inhibition and Disynaptic Group I Inhibition

The lower voluntary activation of muscles during wrist flexion (30°) might be partly related to an increased antagonistic coactivation during MVC of the wrist flexors indicated by an enhanced ECRB·FCR^−1^ ratio. The neural control of the wrist joint muscles differs from that of others, e.g., ankle or elbow joint muscles, since the FCR and ECRB are not strict antagonists and partly act as synergists according to the task requirements. In order to realize these functions recurrent inhibition exists between them which has been found to be amplified by increasing antagonistic muscle activity, respectively (Aymard et al., 1997). Since Renshaw cells of the wrist muscles do not project on group I interneurons, which is for example the case for muscles surrounding the ankle and elbow joint, recurrent inhibition and reciprocal inhibition can be modulated independently. In contrast to other muscles, e.g., ankle or elbow joint muscles, disynaptic group I inhibition of wrist muscles is organized differently with a major contribution of Ib afferents to reciprocal inhibition (Wargon et al., 2006). It is possible that the increased antagonistic coactivity raised the Ib afferent input to the extensor-coupled group I inhibitory interneurons which limited wrist flexor activation. In addition to that, cortical control of the relevant interneurons (Rothwell et al., 1984) might have changed with the wrist joint in flexion (30°) compared with the wrist joint in extension (−30°), thereby altering voluntary activation of the wrist flexors.

In summary, different angle-dependent excitatory and inhibitory inputs to spinal and supraspinal centers might have contributed to the reduced voluntary drive to the wrist flexors and the increased antagonist·agonist^−1^ EMG ratio at a wrist joint angle of 30° compared to −30°. These mechanisms might have operated conjointly in order to limit wrist flexor activation and to enhance the antagonist·agonist^−1^ EMG ratio, probably resulting in an increased wrist joint stiffness.

### Joint Angle Dependence of Unpotentiated and Potentiated Twitch Torques

Although angle-dependent differences in voluntary activation seem to have an effect on the MVT-generating capacity of the wrist flexors, the force-length properties of their MTU are the primary drivers of varying strength capacities at different muscle lengths. *In vivo*, the force-length relationship manifests as the torque-joint angle relationship which also depends on the variable angle-dependent moment arms of the muscle force vectors. As previously shown for other joint complexes (Koh and Herzog, 1995; Rassier et al., 1999; Hansen et al., 2003), we found that the torque-joint angle curve of the wrist flexors shifted to the right for electrically induced submaximal contractions compared to MVCs with the highest unpotentiated peak twitch torques at long MTU length. The same applied to the rate of twitch torque development for the unpotentiated twitch torques. This is likely due to the angle-dependent modulation of the force-length properties of the MTU leading to alterations in actin-myosin interaction (Rassier et al., 1999), the intrinsic muscle properties which are related to Ca^2+^ sensitivity (MacIntosh, 2010), and the series elastic properties (Kawakami et al., 2002). The latter aspect is probably a strong contributor to the decreasing electromechanical delay of the unpotentiated twitch torques from short to long wrist flexors observed in this study. Increasing the length of the MTU leads to a rise in tendon stiffness which optimizes force transmission to the bones. This is especially important for twitch contractions which are characterized by a short duration compared to voluntary or tetanic contractions (Koh and Herzog, 1995). Activity-induced potentiation caused by MVC partly abolished these angle-dependent differences in peak twitch torque, rate of twitch torque development, and electromechanical delay especially for adjacent joint-angles, i.e., flexion vs. neutral (30° vs. 0°) as well as neutral vs. extension (0° vs. −30°). This is mainly due to regulatory light chain phosphorylation which increases mobility of the myosin heads bringing them closer to the thin filaments and thereby increasing the probability of myosin-actin interaction. As described previously for other muscles (MacIntosh, 2010), activity-induced potentiation was greater in shorter than in longer wrist flexors. This is attributed to the fact that muscle lengthening itself reduces the distance between myofilaments so that regulatory light chain phosphorylation becomes less effective (Rassier et al., 1999; MacIntosh, 2010).

The contribution of a second supramaximal electrical stimulus to the unpotentiated peak twitch torque production was analyzed by comparing twitch torques induced by single and paired electrical stimuli. Torque summation was different between joint angles and highest at short muscle length. Similar results have been recently obtained for the plantar flexors and it has been suggested that the slack of the series elastic elements and the length-dependent sensitivity of the contractile elements contribute to this phenomenon (Mayfield et al., 2015). With the wrist joint in a flexed position, the first electrical stimulus has to take up the slack of the MTU. Thus, its contribution to the torque output is impaired, whereas the second electrical stimulus is able to induce a non-linear increase in torque. Due to the lower Ca^2+^ sensitivity at short compared to long muscle length, the contractile elements additionally benefit from the increased Ca^2+^ release and Ca^2+^ summation induced by the second electrical stimulus. Just like for the other twitch torque parameters, activity-induced potentiation reduced these differences between joint angles potentially due to the same mechanisms discussed above.

### Clinical Application

The results of the present study provide valuable information about the contribution of neural and muscular properties to changes in strength capabilities of the wrist flexors at different wrist joint angles. These data could help to understand normal wrist function, which is a first step in determining the underlying mechanisms of musculoskeletal disorders and in giving recommendations for the restoration of musculoskeletal function after injury. Wrist injuries and disorders are common in every age group and are usually fall-, sport-, and work-related (Hill et al., 1998). These musculoskeletal impairments, whether traumatic or overuse induced, translate into reduced range of motion and strength capabilities (Hill et al., 1998; Beumer and Lindau, 2014), which result in severely limited wrist and hand function with negative effects on activities of daily living. Investigating the neural and muscular contributions to these impairments in future studies would help to tailor specific interventions to increase wrist and hand function following traumatic or overuse wrist injuries.

### Limitations of the Study

In the present study, neuromuscular function of the wrist flexors of the left arm was investigated in recreationally active, right-handed, healthy males. Therefore, the present results might not be applicable to left-handed male, female, and older participants. Beyond that, the used techniques did not allow to determine the precise mechanisms behind the observed effects of the wrist joint angle on neuromuscular function of the analyzed wrist flexors. Furthermore, our data are only valid for the neuromuscular function of the wrist flexors innervated by the median nerve and not for those innervated by the ulnar nerve.

## Data Availability

The datasets generated for this study are available on request to the corresponding author.

## Ethics Statement

All procedures performed in studies involving human participants were in accordance with the ethical standards of the institutional and/or national research committee and with the 1964 Helsinki declaration and its later amendments or comparable ethical standards. Informed consent was obtained from all individual participants included in the study.

## Author Contributions

MB and FH designed the study and analyzed the data. MB, FH, AM-M, and JS collected the data. MB wrote the manuscript. FH, AM-M, JS, E-MR, and VZ contributed to writing, reviewing, and editing of the manuscript. All authors approved the final version of the manuscript.

### Conflict of Interest Statement

The authors declare that the research was conducted in the absence of any commercial or financial relationships that could be construed as a potential conflict of interest.
